# Single-cell protein profiling in microchambers with barcoded beads

**DOI:** 10.1038/s41378-019-0099-5

**Published:** 2019-11-04

**Authors:** Lucas Armbrecht, Rafael Sebastian Müller, Jonas Nikoloff, Petra Stephanie Dittrich

**Affiliations:** Department of Biosystems Science and Engineering, ETH Zurich, Mattenstrasse 26, 4058 Basel, Switzerland

**Keywords:** Engineering, Chemistry

## Abstract

Single-cell profiling provides insights into cellular behaviour that macroscale cell cultures and bulk measurements cannot reveal. In the context of personalized cancer treatment, the profiling of individual tumour cells may lead to higher success rates for therapies by rapidly selecting the most efficacious drugs. Currently, genomic analysis at the single-cell level is available through highly sensitive sequencing approaches. However, the identification and quantification of intracellular or secreted proteins or metabolites remains challenging. Here, we introduce a microfluidic method that facilitates capture, automated data acquisition and the multiplexed quantification of proteins from individual cells. The microfluidic platform comprises 1026 chambers with a volume of 152 pL each, in which single cells and barcoded beads are co-immobilized. We demonstrated multiplexed single-cell protein quantification with three different mammalian cell lines, including two model breast cancer cell lines. We established on-chip immunoassays for glyceraldehyde-3-phosphate dehydrogenase (GAPDH), galectin-3 (Gal-3) and galectin-3 binding protein (Gal-3bp) with detection limits as low as 7.0 × 10^4^, 2.3 × 10^5^ and 1.8 × 10^3^ molecules per cell, respectively. The three investigated cell types had high cytosolic levels of GAPDH and could be clearly differentiated by their expression levels of Gal-3 and Gal-3bp, which are important factors that contribute to cancer metastasis. Because it employed commercially available barcoded beads for this study, our platform could be easily used for the single-cell protein profiling of several hundred different targets. Moreover, this versatile method is applicable to the analysis of bacteria, yeast and mammalian cells and nanometre-sized lipid vesicles.

## Microfluidics: Identifying and quantifying a single cell’s proteins

An assay that traps cells in micrometre-sized chambers is able to identify and quantify a single cell’s proteins. This technique could prove beneficial in situations such as cancer, in which elucidating the variation between tumour cells could improve pharmacological strategies. Identifying and quantifying the proteins of a single cell has been a challenge for researchers, leading Petra Dittrich and her team from Switzerland’s ETH Zurich to develop their system which traps antibody-based assays bound to tiny barcoded beads in microfluidic chambers with single cells. The team identified and quantified three different proteins, two of which are implicated in cancer metastasis, from three different mammalian cell lines. Their device had very low detection limits and can work with several hundred different targets and be used with bacteria, yeast, mammalian cells and lipid vesicles.

## Introduction

Cell-to-cell heterogeneity is a universal characteristic of cell populations, allowing essential processes such as cell adaptation and evolution^1^. Cellular heterogeneity plays an important role in diseases such as cancer, in which the presence of cancer cells of various subtypes can influence the progress of the disease and therapeutic success. When cellular variations in tumour tissue or between different metastatic sites can be precisely determined, the best treatment for each patient can be selected at an early stage of the disease^2^. In recent years, cancer immunotherapies have been introduced that provide improved success rates and reduced side effects compared with traditional chemotherapy in certain subsets of patients^3,4^. As immunotherapies target specific biomolecules, they require detailed profiling of the patient’s tumour phenotype to select the correct drug. Currently, most antibody-based therapies target membrane receptors such as human epidermal growth factor receptor 2 (HER-2), which is upregulated in approximately one in four breast cancer patients^5^. In addition, secreted or intracellular factors such as vascular endothelial growth factor (VEGF) have also been found to be suitable drug targets for therapy^6^. Many new immunotherapeutic drugs are currently being tested, and numerous molecules have been suggested as potential targets^7^. Prominent examples are galectin-3 (Gal-3) and galectin-3 binding protein (Gal-3bp), which are actively involved in cancer metastasis^8–11^. However, the analysis of secreted or intracellular proteins at the single-cell level remains difficult. Nonetheless, such an analysis would certainly improve the success rates of cancer therapies, as it complements existing tools used for nucleic acid sequencing studies^12,13^.

Recently, microfluidic devices have received increasing attention in the research community as a result of their ability to elucidate cellular heterogeneity, monitor drug responses and quantify biomolecules from individual cells^14,15^. Microfluidic devices provide unique features that are useful for these applications, as they allow the manipulation of individual cells and the confinement of cells in microcompartments for subsequent analysis^16–18^. For cell capture, several solutions have been introduced in the past, such as hydrodynamic traps and wells^19–21^, in which cells are captured passively based on size. Alternative techniques use di-electrophoretic^22^, acoustophoretic^23^, or optical forces^24^, which allow for capturing and releasing cells on demand. For many applications, capturing cells according to phenotypic characteristics, e.g., surface markers, is required. This was successfully performed by using magnetic trapping, in which antibody-coated magnetic beads bind selectively to the surface proteins of cells. For example, Saliba et al. were able to capture cancer cells with self-assembled arrays of magnetic beads very efficiently. Afterwards, they characterized the membrane proteins on the captured cells with immunohistochemistry by adding fluorescently labelled antibodies^25^.

Beyond the utilization of immunostaining, the ultimate goal is the simultaneous analysis of multiple secreted or intracellular targets at a single-cell level. Only a few recent studies have demonstrated such multiplexed analysis by employing immunoassays with surface-bound antibodies^26–28^. However, such strategies require that the capture antibodies be immobilized on the microfluidic chip prior to the experiment, which limits flexibility, increases processing time, and often leads to background signal when unprocessed sample makes contact with the dedicated capture sites. To overcome these drawbacks, microspheres with unique fluorescent labels can be used as mobile substrates for the assay of choice^29–31^ and have already been used for multiplexed measurements on microwell devices^32^. These barcoded beads together with microfluidic methods for cell capture may facilitate single-cell protein profiling.

To achieve this goal, our group developed several microfluidic devices for single-cell analysis in recent years, and we achieved extraordinarily high limits of detection by the implementation of immunoassays for a single selected target. The integration of round valves was essential to achieve such high sensitivities. The valves isolate cells in individual small reaction chambers and enable efficient target capture and the precise control of assay steps such as cell lysis, washing, immune labelling and enzymatic reactions^33,34^.

Here, we introduce a new, versatile combination that utilizes magnetic cell trapping with specific cell-membrane markers and highly parallel multiplexed proteome analysis. This was achieved with bead-based immunoassays for the analysis of cancer cells. The microfluidic platform comprised more than 1000 isolated microfluidic compartments based on doughnut-shaped valves (hereafter called microchambers) designed to co-capture cells and barcoded beads in capture wells located in the centre of each microchamber. For simplified data analysis, we developed an intuitive software tool to process large multidimensional image data sets written in MATLAB. With this system, we conducted multiplexed immunoassays to determine intracellular glyceraldehyde-3-phosphate dehydrogenase (GAPDH), galectin-3 (Gal-3) and galectin-3 binding protein (Gal-3bp) levels in three different mammalian cell lines with detection limits down to 1.8 × 10^3^ molecules.

## Results

### Chip design and operation

**Fig. 1:**
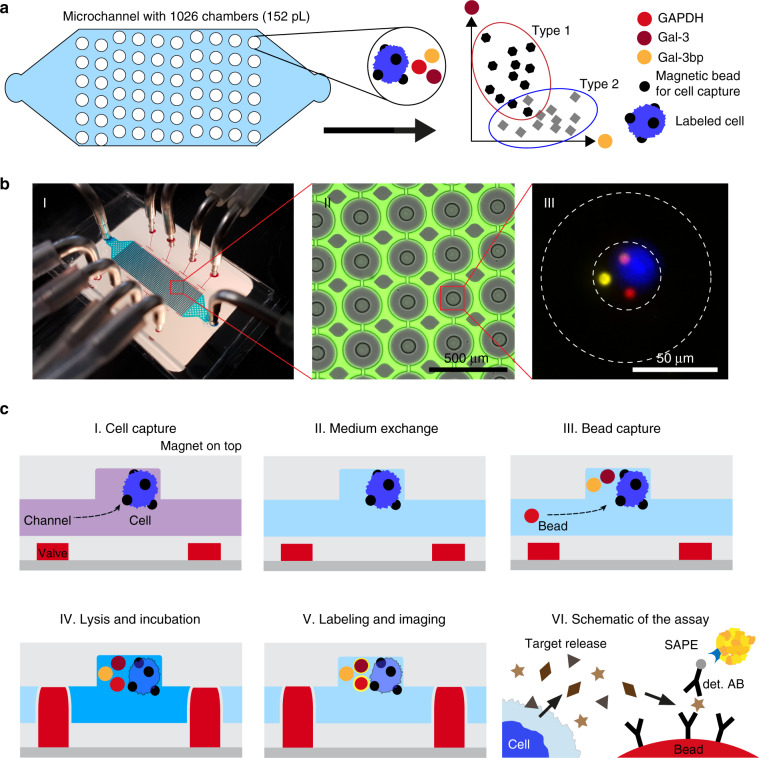
Microfluidic chip design and functionality for single-cell protein profiling. **a** Schematic of the microfluidic chip with 1026 individual microfluidic chambers used for single-cell analysis. Protein quantification was performed by immunoassays with fluorescently barcoded beads, which were co-immobilized with the cells of interest. **b** Images of the chip and microchambers. (I) Photograph of the chip with the tubing used for the supply of the liquids (blue) as well as the eight control lines (red) used to activate the valves. The permanent magnet that is positioned on top of the chip during the entire experimental procedure is not shown. (II) Micrograph of the chamber array depicting the actuated round valves in grey, as well as the surrounding fluorescent solution (green). The columns between the microchambers (in grey) were used to stabilize the microchannel. (III) A composite fluorescent image of one chamber holding a single cell (blue colour) and three barcoded beads (yellow, red and pink colour) for multiplexed immunoassays. **c** The protocol used for sensitive proteomic profiling included (I) cell capture, (II) medium exchange, (III) co-capture of beads, (IV) cell lysis and incubation, (V) fluorescent labelling and final imaging. (VI) The immunoassays with the beads required a two-step labelling procedure, first with the biotinylated detection antibodies (det. AB) and subsequently with streptavidin-PE (SAPE)

The design of our microfluidic device is shown in Fig. 1. It facilitates the selective capture of magnetically labelled cells together with barcoded beads in one of >1000 capture wells. Both the beads that bind to the cells and the barcoded beads are superparamagnetic. Thus, they are pulled up into the capture well when a permanent magnet is placed on top of the microfluidic device. The captured cells are isolated in a tiny reaction chamber to allow the analysis of each individual cell. The process used for any immunoassay includes cell lysis, incubation and washing steps, and imaging. Cell isolation is accomplished with integrated doughnut-shaped valves that enclose the cells in a microchamber with a volume of 152 pL upon actuation at a pressure of ≥2 bar (see Figs. S1 and S2). The functionality of the device, its operation and the software used for data analysis are described in detail in the electronic supplementary information.

#### Bead capture

**Fig. 2:**
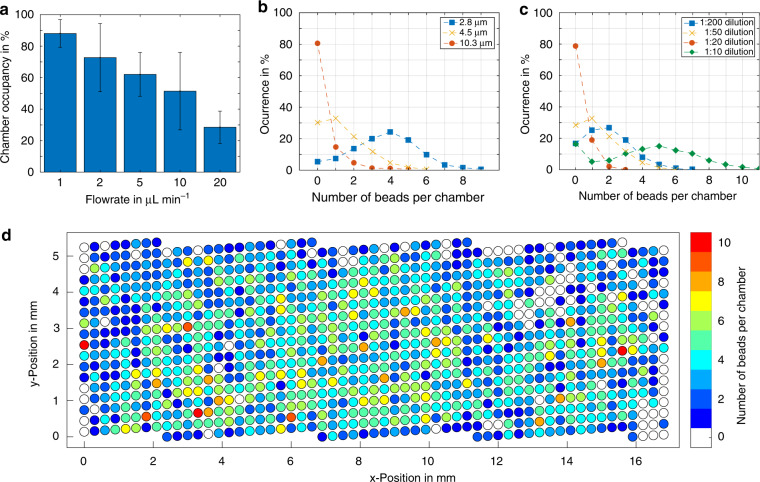
On-chip capture of magnetic beads. **a** Chamber occupancy with at least one magnetic bead (diameter of 4.5 µm) with varying flow rates. **b** Number of particles captured per chamber for different bead sizes at a flow rate of 2 µL min^−1^ and 1:50 dilution (concentration of 20 µg mL^−1^). **c** Distribution of particles per chamber at different dilution rates (constant flow rate 2 µL min^−1^, concentration 20 µg mL^−1^, bead size 4.5 µm). **d** Distribution of captured particles across the chip for 4.5 µm beads at a flow rate of 2 µL min^−1^ and a dilution of 1:50

To determine the best conditions for bead capture, we performed experiments with 4.5 µm magnetic beads using flow rates between 1 and 20 µL min^−1^ (see Fig. 2a). Flow rates below 1 µL min^−1^ resulted in the undesired immobilization of beads on the ceiling of the channel, as the attractive magnetic forces exceeded the fluidic drag forces. As a measure of capture success, we determined the fraction of occupied traps on the chip. At an optimal flow rate of 1 µL min^−1^, we found that more than 88% of all chambers were occupied with at least one bead. Next, we performed experiments with magnetic beads of sizes ranging from 1 to 10 µm. As the magnetic moment of a bead scales with its volume ($\propto d^3$) while the fluidic drag forces scale with the cross-sectional area ($\propto d^2$), larger beads can be captured efficiently at higher flow rates^35^. However, most available magnetic bead suspensions have similar material concentrations of ~1 mg mL^−1^. Therefore, suspensions of larger beads contain fewer particles per volume of fluid than suspensions of smaller particles. This effect overcompensates for the higher magnetic moments of larger particles. Hence, we observed a decrease in the chamber occupancy according to the bead size under similar experimental conditions (same dilution, flow rate and capturing time, see Fig. 2b, c). By determining the fraction of immobilized beads in the capture sites compared with the total number of beads that were used for each test, we observed a capture efficiency of 11.3 ± 3.0% regardless of the dilution factor of the 4.5 µm beads. When we added the same total number of differently sized beads to the chip, we observed no clear relationship between bead size and the bead capture distributions on the platform (see Fig. S3). Furthermore, we observed higher optical transparencies for the 6.5-µm magnetic barcoded Luminex beads, which indicated a low magnetic nanoparticle content. As a result, the optimal flow rate for bead capture dropped to 1 µL min^−1^ (see Fig. S4). In all experiments, we observed that a greater number of beads were trapped in the central wells of the chip compared with the wells near the wall or at the channel entrance or exit. We suspect that small magnetic field inhomogeneities and minor differences in the flow velocity (see Figs. S5, S6, and S7) were responsible for this effect (see Fig. 2d).

#### Cell capture

**Fig. 3:**
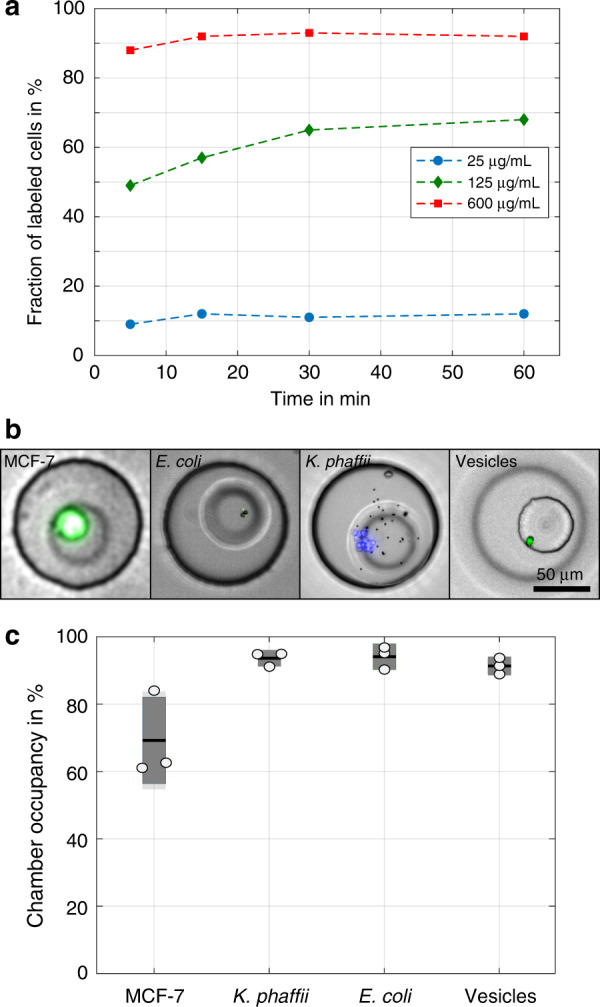
Cell capture in microchambers. **a** Labelling of MCF-7 cells with functionalized beads (diameter: 4.5 µm). A successfully labelled cell binds to at least one magnetic bead. The labelling of >90% of beads was achieved with an incubation time of at least 15 min at a concentration of 600 µg mL^−1^. **b** The use of beads functionalized with specific antibodies allowed for the capture of mammalian (MCF-7), yeast (*K. phaffii*) and bacterial cells (*E. coli*) as well as lipid vesicles, and (**c**) larger target organisms yielded lower capture success

The device facilitated the capture of various cell types and vesicles that were bound to magnetic beads. To trap mammalian cells of epithelial origin, such as MCF-7 cells, magnetic beads were functionalized with anti-epithelial cell adhesion molecule (EpCAM) antibodies. We found that a 600 µg mL^−1^ bead concentration yielded labelling efficiencies above 90% with an incubation time of at least 15 min (see Fig. 3a). We also functionalized magnetic beads with antibodies to target surface markers of *Escherichia coli (E. coli)* and *Komagataella phaffii (K. phaffii)* and biotin-PEG-cholesterol to bind large unilamellar vesicles ≤200 nm in size generated by extrusion (see Fig. 3b)^36^. The capture of *E. coli* and *K. phaffii* yielded high chamber occupancies above 90%, while the capture of MCF-7 cells yielded mean chamber occupancies of 69.2% and a capture efficiency of ~18% (Fig. 3c). The main reason for this difference was the large size of MCF-7 cells, which resulted in higher fluidic drag forces. In addition, MCF-7 cells are an adherent cell line, so they tend to form cell clusters during the labelling procedure and have decreased cell densities after cultivation. Similar capture efficiencies to those observed for MCF-7 cells were observed for HEK-293T and SK-BR-3 cells (69.1% and 63.0%, respectively). Due to the large number of microchambers, more than 600 tests in parallel can be performed on one device even at 60% chamber occupancy. For MCF-7 cells, we found that approximately one-third of all trapping sites were filled with a single cell and one in five with two cells, whereas for smaller cell types that grow in clusters, such as *K. phaffii*, a larger heterogeneity in cell number per trapping site was observed (Fig. S8).

### Barcoded immunoassays

**Fig. 4:**
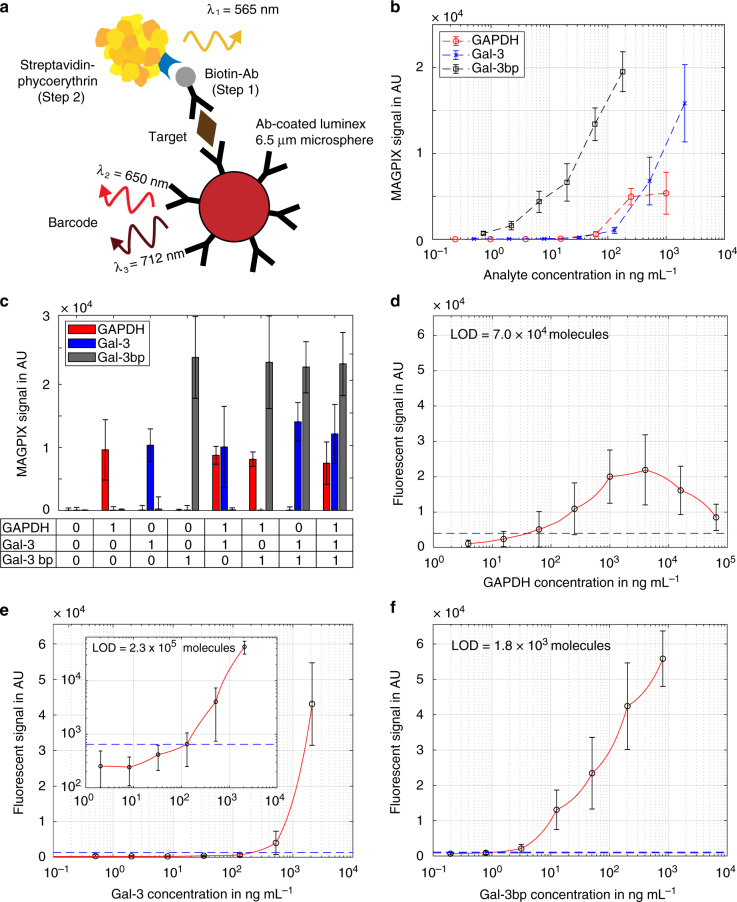
Immunoassays with barcoded Luminex beads. **a** Schematic illustration of the immunoassay on the bead surface. Labelling was conducted in two steps with biotinylated antibodies (step 1) and streptavidin-conjugated Phycoerythrin (step 2). (**b**) Calibration of GAPDH, Gal-3 and Gal-3bp in a 96-well plate format. **c** Multiplexing with all three targets revealed that there was no cross-reaction between the assays. The digital combinations of the three targets are noted underneath the table, with (“1”) indicating the presence and (“0”) indication the absence of the analyte (**d**–**f**). On-chip calibration curves in microchambers with the respective limits of detection (LODs). The blue dotted lines represent the corresponding background signals plus three times the standard deviation. The red lines represent the linear interpolation fit (please note the semi-logarithmic *x*-axis scaling that results in the nonlinear appearance of the linear interpolation curves)

Luminex ProcartaPlex assays (see schematic in Fig. 4a) of GAPDH, Gal-3 and Gal-3bp were first performed off-chip with a Magpix reader (Luminex Corporation, Austin, Texas, USA). We acquired calibration curves with the protein standards for GAPDH, Gal-3 and Gal-3bp in 96-well plates. The lower limits of quantification for the three assays were given by the manufacturer as 0.2, 2.0 and 0.2 ng mL^−1^, respectively. However, these values can only be achieved when at least 100 beads contribute to each measurement. When signals from individual beads are used for the assay, the standard deviation increases, and the limits of detection (LODs, defined as the mean plus three times the standard deviation of the background signal) rise to 8, 25 and 2 ng mL^−1^ for GAPDH, Gal-3 and Gal-3bp, respectively (Fig. 4b). Compared with the Gal-3 and Gal-3bp assays, the GAPDH assay showed significantly higher bead-to-bead variation and lower overall signal in both well plate format and on the microfluidic device, which prevented quantitative measurements. Nonetheless, we confirmed the high specificity of all antibodies for the respective compounds. The three assays could therefore be performed in parallel without notable cross-reactivity (Fig. 4c).

Once the off-chip assays were established, we successfully performed the complete on-chip assays in the 152 pL-sized microchambers. To do so, we used a fluorescence microscope for the detection of the fluorescent signal (see Fig. 4d–f). With this setup, we determined LODs of 43.1 ng mL^−1^ or 7.0 × 10^4^ molecules for GAPDH, 19.6 ng mL^−1^ or 2.3 × 10^5^ molecules for Gal-3, and 1.2 ng mL^−1^ or 1.8 × 10^3^ molecules for Gal-3bp. Due to strong differences in the signal intensities and nonlinear responses of the three assays, we used interpolation to estimate the LODs. For GAPDH, we observed a decreasing signal instead of the expected plateau for concentrations >5 µg mL^−1^. This behaviour produces similar outcomes as the high-dose Hook effect and is related to the binding of GAPDH to the channel walls at high concentrations. Therefore, the quantification of GAPDH was not possible.

### Multiplexed single-cell analysis

**Fig. 5:**
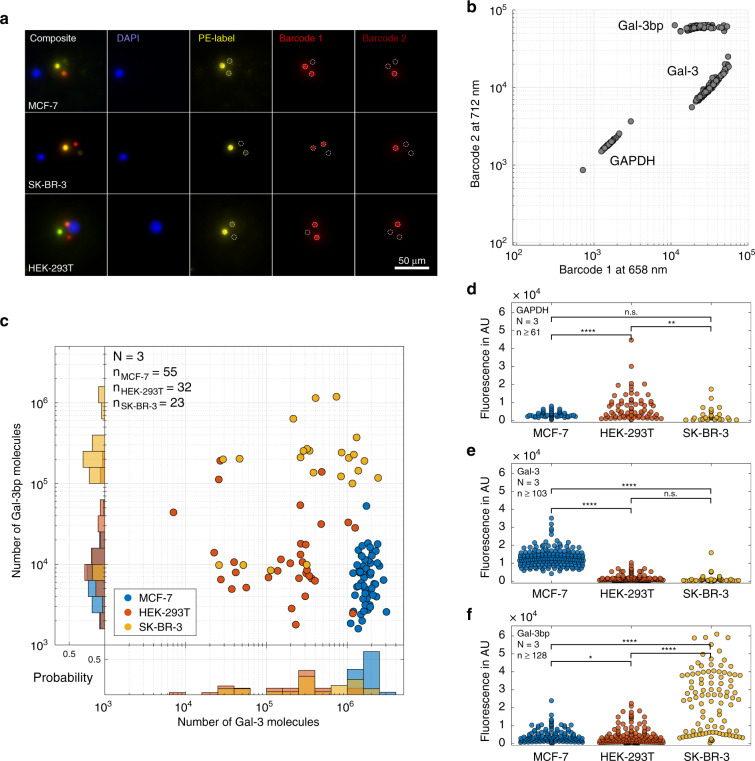
Multiplexed single-cell analysis. **a** Fluorescent images of selected microchambers that show the acquired images prior to analysis. Cells were visualized by staining of the nuclei, while the barcoded beads were identified by the ratio of emission at 658 and 712 nm. **b** Based on the fluorescent signals of the two barcoded labels, the bead identity could be determined. **c** Profiles of Gal-3 and Gal-3bp in the three investigated cell types (depicted in different colours). The data were derived from microchambers in which both types of functionalized beads (Gal-3 and Gal-3bp) were co-immobilized with one individual cell. With few exceptions, each cell type exhibited a clear expression pattern for the two proteins. **d**–**f** Fluorescence signals derived from the functionalized beads for all tested cells. Here, we used microchambers in which at least one type of barcoded bead was co-captured. While SK-BR-3 cells expressed higher Gal-3bp levels than HEK-293T and MCF-7 cells, the latter showed stronger Gal-3 expression when compared with HEK-293T and SK-BR-3 cells. For GAPDH (**d**), we observed an effect similar to the high-dose Hook effect, resulting in a signal decrease at increasing target concentrations (Fig. 4d). Hence, HEK-293T cells, which showed the highest fluorescence in the GAPDH assay, actually had lower GAPDH concentrations than the other two cell lines; significance levels are indicated with n.s. *P* > 0.05, *P ≤ 0.05, **P ≤ 0.01, ***P ≤ 0.001 and ****P ≤ 0.0001

Once the immunoassays were established on-chip, we combined magnetic cell capture with the capture of barcoded Luminex beads for the analysis of the three proteins in two breast cancer cell lines (MCF-7 and SK-BR-3) and a standard model cell line (HEK-293T). After capturing the cells, we added the mixture of the three bead types, which were then dispersed stochastically within the capture sites. As a result, 13.8% of chambers were occupied with all three types of beads, another 49.2% of the chambers contained two types of beads, and 26.1% of the chambers contained only one type of bead. Consequently, all three targets were on average measured in no more than 38 chambers that contained single cells per chip. This fact represents the main limitation of the current platform. However, any two targets were simultaneously assayed from the measurement of 164 single cells per chip. In addition, the intracellular content of each individual target was determined in several hundred chambers on a single microfluidic chip. Figure 5a shows images in which a cell and three different types of beads were captured together in one microchamber. The imaging of the fluorescence intensity at different emission wavelengths enabled the clear distinction of the cells and the barcoded beads, and the quantification of the target proteins. The intensities of the fluorescence signals *I*_λ=658nm_ and *I*_λ=712nm_ allowed for the unambiguous assignment of the bead types (Fig. 5b), whereas nuclear staining (DAPI) was used to identify the cells. The intensity of the phycoerythrin fluorescence that was co-localized with the fluorescence of the beads enabled the quantification of the target proteins. Notably, the use of barcoded beads as assay carriers permitted the addition of the antibody-coated substrate beads after cell capture and subsequent washing. As a result, the background signal was reduced by a factor of three (see Fig. S9).

Using this procedure, we performed the multiplexed analysis of GAPDH, Gal-3 and Gal-3bp expression on MCF-7, HEK-293T and SK-BR-3 cells captured in the microfluidic chambers. After capturing cells from any of the three tested cell lines, lysis buffer (0.25% [v/v] Triton X-100 in PBS) was introduced into the chip, and the chambers were opened for ~1 s to add the lysis buffer into the microchambers. The fast closure of the valves ensured that the released intracellular compounds remained in the microchambers during the subsequent cell lysis and bound to the beads during the incubation. We analysed chambers with all three types of beads (Fig. 5c) as well as chambers that contained only one or two types of beads (Fig. 5d, e). The observed Gal-3 and Gal-3bp expression patterns revealed clear differences between the three cell types (Fig. 5c). Using the on-chip calibration for the corresponding immunoassays, we found average intracellular levels of 1.8 × 10^6^ molecules for Gal-3 and 9.3 × 10^3^ molecules for Gal-3bp in MCF-7 cells. Compared with MCF-7 cells, the breast cancer cell line SK-BR-3 showed elevated intracellular levels of Gal-3bp (0.2 × 10^6^ molecules per cell) with a high cell-to-cell variation and reduced levels of Gal-3 (0.6 × 10^6^ molecules per cell). Finally, the HEK-293T cells were found to express low quantities of both Gal-3 and Gal-3bp (0.3 × 10^6^ molecules per cell and 1.8 × 10^4^ molecules per cell, respectively). Notably, the population averages of our results were in line with previously reported data from bulk RNA sequencing experiments (available from www.proteinatlas.org, see Table S1).

The fluorescent signals for the GAPDH assay in single HEK-293T cells were significantly higher than those in MCF-7 and SK-BR-3 cells. Although the quantification of the GAPDH signals was not possible, the results were in good agreement with previously reported RNA sequencing data, as the GAPDH expression levels were expected to be above 5 µg mL^−1^. The GAPDH signal was correlated inversely with the fluorescence signal of the assay due to the non-specific adsorption of GAPDH to the channel surfaces at high concentrations (Figs. 4d and S10). As a result, the detection antibody bound to these surface-bound molecules, thereby preventing sandwich formation at the beads, which resulted in decreased fluorescence from the beads. This behaviour is similar to the high-dose Hook effect^37^. The antigen-saturated detection antibodies in solution were then washed off in subsequent washing steps, resulting in a misleadingly low signal for GAPDH. By combining the measurements of all targets, we observed that the resulting expression patterns of the three cell lines were clearly distinct (Fig. 5d–f). Altogether, our system was found to be well suited to directly assess the expression levels of the important metastasis-related proteins Gal-3 and Gal-3bp at the single-cell level.

## Conclusion

In conclusion, we developed a versatile microfluidic platform for the parallel analysis of single cells in 1026 microchambers, which each had an internal volume of only 152 pL. The co-capturing of bead-bound cells and barcoded beads for immunoassays in microfluidic chambers was based on magnetic forces and enabled sensitive and multiplexed analysis of proteins. The platform was supported by an intuitive software tool that allowed us to extract all the necessary information from multidimensional imaging data. We successfully determined the expression patterns of GAPDH, Gal-3 and Gal-3bp in three different cell lines (MCF-7, HEK-293T and SK-BR-3) and could clearly discriminate these cell populations. Both MCF-7 and HEK-293T cells revealed lower expression levels of Gal-3bp compared with the SK-BR-3 cells, and we observed lower Gal-3 levels both in HEK-293T and SK-BR-3 cells than in MCF-7 cells. As Gal-3 and Gal-3bp are both involved in the metastatic process and were recently proposed as biomarkers^38,39^, we are confident that the presented system is an important step towards the phenotypic analysis of patient-derived tumour cells, which is necessary for the success of personalized cancer immunotherapies.

Currently, the main limitations of the presented platform are the low throughput and the difficulties related to the non-specific adsorption of GAPDH to the polydimethylsiloxane (PDMS) chip surfaces. Improved surface blocking agents and washing protocols for this device will be helpful for future studies. Alternatively, the use of a different chip material than PDMS, which is known to absorb small hydrophobic molecules, would be beneficial. We will also focus on improving the cell capture efficiency to enable the analysis of circulating tumour cells from liquid biopsies. By taking advantage of the numerous commercially available bead-based immunoassays, the simultaneous measurement of metabolites, secreted molecules or microRNAs is possible. As bead-barcoding technology provides 50 unique barcodes, highly multiplexed measurements are possible and might reveal deeper insights into intracellular processes and signalling pathways in cancer cells^40^. In addition, the platform could be employed to study yeast, bacteria or cell-mimicking vesicles, which may result in additional applications in the future.

## Materials and methods

### Device fabrication

The microfluidic devices were made from PDMS by soft lithography. Two layers of PDMS, a so-called fluid layer and a control layer, are required for the final device. The two corresponding silicon master moulds were fabricated using standard soft lithographic methods with SU-8 3025 negative photoresist (MicroChem Corp., Westborough, MA, USA) (see the supplementary information for detailed information). Both layers were fabricated by mixing the PDMS oligomer and the curing agent (Sylgard 184 silicone elastomer kit, Dow Corning, Midland, MI, USA) at a ratio of 10:1 (see Fig. S11). Next, the mixture was degassed for 15 min, and 40 g PDMS was poured onto the master mould to generate the fluid layer and cured at 80 °C for 2 h. In parallel, ~5 g of the PDMS mixture was spin-coated at 3200 rpm for 60 s onto the master mould to generate the control layer and baked at 80 °C for 1 h. The spin-coating process generates an ~30 μm-thick PDMS layer that covers the 20 μm high pressure control. Afterwards, the flow layer was peeled off the master mould and cut to size, and the fluid inlet and outlet ports were punched with a 1.5 mm biopsy puncher (Integra Miltex, York, PA, USA). To bond the two PDMS parts, 1 mL PDMS curing agent was spin-coated onto a blank 4″ silicon wafer for 40 s at 6000 rpm. The fluid layer was pressed onto the coated wafer to transfer a small amount of the curing agent to the PDMS surface and then manually aligned on top of the fluid layer. Afterwards, the chip assembly was incubated for 30 min at room temperature and subsequently baked for 2 h at 80 °C to bond the two layers. Next, the assembled PDMS chip was peeled from the control master, and the ports for the pressure valves were punched using a 1 mm biopsy puncher (Integra Miltex, York, PA, USA). After exposure to oxygen plasma for 120 s in a PDC-32G plasma cleaner (Harrick Plasma, Ithaca, NY, USA) at maximum power (18 W), the PDMS chip was finally bonded to a 50 mm × 24 mm glass coverslip (thickness: #3, Menzel, Braunschweig, Germany) and placed on a hotplate for 2 h at 80 °C.

### Magnetic bead functionalization

Commercial streptavidin-coated magnetic particles were purchased with diameters of 1 μm, 2.8 μm (both from Dynabeads, Thermo Fisher Scientific, Waltham, MA, USA), 4.5 μm and 10.4 μm (both from Microparticles GmbH, Berlin, Germany). In addition, 6.5 μm barcoded pre-functionalized beads and assay reagents for the detection of GAPDH, Gal-3 (all ProcartaPlex Simplex kits, Thermo Fisher Scientific, Waltham, MA, USA) and Gal-3bp (Bio-Techne AG, Zug, Switzerland) were obtained. To allow for selective cell labelling, magnetic particles of different sizes were washed three times on a custom magnetic rack in 10 mM phosphate-buffered saline (PBS, Sigma-Aldrich, St. Louis, MO, USA) and diluted to a final concentration of 0.2 mg mL^−1^. The beads were then incubated with 100 µL of 5 µM biotin-Atto565 (Sigma-Aldrich, St. Louis, MO, USA) or 100 µL of 50 µg ml^−1^ species-specific biotinylated antibody in 10 mM PBS (see Table 1) for 45 min with constant rotation on a MACSMIX rotary shaker (Miltenyi Biotec, Bergisch Gladbach, Germany). After incubation, the beads were washed six times with 10 mM PBS. Finally, the beads were transferred to a new Eppendorf tube (Eppendorf Protein low-bind tubes, Thermo Fisher Scientific, Waltham, MA, USA) and stored at 4 °C in PBS supplemented with 1% [w/w] bovine serum albumin (BSA, heat shock fraction, Thermo Fisher Scientific, Waltham, MA, USA) until use.

**Table 1:** Bead functionalization with biotinylated antibodies for the specific capture of MCF-7, HEK-293T, SK-BR-3, *K. phaffii*, and *E. coli* cells and large unilamellar vesicles

Target	Capture molecule	Cell stain
MCF-7, HEK-293T, SK-BR-3	Anti-EpCAM-biotin	Calcein AM
*K. phaffii*	Anti *K. phaffii*, biotin	Calco-Fluor white
*E. coli*	*E. coli* serotype O/K polyclonal antibody, biotin	Cytosolic GFP
Vesicles (LUVs)	Biotin-PEG-cholesterol	Incorporated calcein

### Measurement setup

The experiments with the microfluidic chip platform were conducted on a fully automated inverted Nikon Ti2 epifluorescence microscope (Nikon Corporation, Tokio, Japan) equipped with an incubation chamber (with CO_2_, humidity, and temperature control). All images were acquired using a ×20 objective (NA = 0.75) and an Orca-Flash 4.0 Scientific CMOS camera with 2044 × 2048 pixels (Hamamatsu, Japan). The transmitted light was generated by an LED light system (CoolLED Ltd, Andover, UK), whereas the fluorescent illumination was provided by a Spectra X LED system (Lumencor, Inc., Beaverton, OR, USA). For the detection of the Luminex barcodes, two dedicated bandpass filter sets (670 ± 30 and 725 ± 40 nm) were employed.

Before each experiment, the microfluidic chip was filled by inserting pipette tips with 20 µL milliQ water into each pressure and fluidic port. The air was removed from the channels by centrifugation for 10 min at 800 RCF. The eight pressure ports on the chip were then connected to a pressure control unit, and the chip was fixed onto the custom microscope stage. The fluidic outlet was connected to a 1 mL plastic syringe with 1/16″ PTFE tubing and a curved metal pin. Finally, the plastic syringe was mounted onto a Nemesys syringe pump (Cetoni GmbH, Korbußen, Germany), and the pump module and the microscope were controlled with a desktop computer. The automated microscope was handled by using the Nikon NIS Elements V 5.02 imaging software (Nikon, Tokyo, Japan), and the syringe pumps were controlled with Nemesys software (Cetoni GmbH, Korbußen, Germany). A 3D printed magnet holder (see Fig. S12) with a 20 × 10 × 5 mm^3^ permanent magnet (#Q-20-10-05-N, Webcraft AG, Uster, Switzerland) was then placed above the chip to maintain the optimal 9 mm distance between the channel and the magnet and was used for all of the following experiments^35^. This distance was determined with fluidic and magnetic field simulations (see Fig. S7).

### Bead capture

Fluorescently labelled superparamagnetic beads were captured at different flow rates of up to 20 μL min^−1^ on the microfluidic chip (information on bead functionalization can be found in the ESI). A 6 μL bead solution was aspirated into a pipette tip that was connected to the chip outlet port (see Figs. S12 and S13). Subsequently, the bead suspension was aspirated from the inlet at a flow rate of 10 μL min^−1^ for 60 s. Next, the magnetic holder was placed above the chip, the flow was reversed and set to the desired flow rate, and 20 μL of the bead suspension was flushed through the channel. The magnetic field pulled the beads towards the ceiling of the microfluidic channel and into the magnetic capture sites. After bead capture, the valves were pressurized to 2 bar to isolate the individual microchambers from the channel (see Fig. S1). Finally, the magnet was removed, the unbound beads were flushed out of the channel at a flow rate of 20 μL min^−1^ for 60 s, and a series of images of the chip was acquired.

### Cell capture

Once the cells of interest were harvested from culture (details in the supplementary information), they were incubated with 4.5 µm pre-functionalized magnetic beads. Then, 20 μL of the functionalized bead solution at a concentration of 1 mg mL^−1^ was transferred to a new Eppendorf tube and placed on the magnetic washer, and the supernatant was removed. Next, 100 μL of cell solution was added to the beads, and the solution was incubated on a rotary shaker for 30 min at the optimal culture temperature for each cell type. Immediately afterwards, 10 μL of sample was introduced into the chip at a flow rate of 10 μL min^*−*1^. Then, the permanent magnet was placed on top of the chip, and the sample was flushed through the channel and washed with 10 μL of washing buffer (PBS with 1% [w/w] BSA) at a flow rate of 0.5 μL min^−1^ as described above. After washing, all valves were pressurized, the magnet was removed, and all microchambers were imaged.

### Bead-based on-chip immunoassays

Immunoassays were performed with 6.5-μm barcoded magnetic Luminex beads to detect GAPDH, Gal-3 and Gal-3bp. A pipette tip with 6 μL bead solution was connected to the chip outlet, and 10 μL of bead solution was aspirated into the channel at 20 μL min^−1^. Next, the magnetic holder was placed above the chip, and the barcoded beads were captured at a flow rate of 1 μL min^−1^ before the pneumatic valves were actuated. Afterwards, the target molecule solution was introduced into the chip at a flow rate of 1 μL min^−1^, and the first set of chambers was opened for ~1 s to exchange the solution in the chamber. Once the corresponding pneumatic valve was closed, the procedure was repeated for a different set of microchambers with a different target concentration. This was followed by a 60 min incubation period, during which the surrounding channels in the chip were constantly flushed with PBS with 1% BSA at a flow rate of 1 μL min^−1^. After incubation, the chip was filled with biotinylated detection antibody (10-fold diluted in Ab diluent provided in the Luminex bead kit), the valves were opened, and the beads were incubated with the antibody mixture for 30 min. Finally, streptavidin-PE (SAPE) labelling solution was introduced into the channel and incubated for 60 min. The beads were finally washed with washing buffer for 30 min at 1 μL min^*−*1^, and imaging was performed.

### Multiplexed protein analysis of single cells

For the protein profiling of single mammalian cells, cell trapping was followed by the capture of 6 μL of antibody-coated barcoded beads as described above. For the multiplexed measurements, Gal-3bp, Gal-3 and GAPDH beads were mixed in a ratio of 1:1:1 [v:v:v]. Next, cell lysis was induced by flushing lysis buffer (0.25% Triton X-100 in PBS) into the channel and subsequently opening the pneumatic valves for ~1 s (see Supplementary Video SV1). After a 60 min incubation, the pressure in the valves was reduced to 1 bar (partially opened) to allow for the exchange of solutions inside the microchambers. This ensured that no cells or beads could escape the microchambers during the labelling procedure. Then, a biotinylated antibody labelling mixture (final dilution of each antibody was 1:10 [v:v]) was added to the chambers and incubated for 30 min. After subsequent washing, the beads were incubated with SAPE solution for another 60 min, washed, and finally imaged with an automated wide-field microscope. Our custom-made image analysis software and procedure is described in the supplementary information.

## Supplementary information

Video 1

CAD device design

Supplemental Material File
