# Supplementary material for: Single-cell protein profiling in microchambers with barcoded beads
Source: Microsyst Nanoeng. 2019 Nov 4;5:55. doi: 10.1038/s41378-019-0099-5 (PMC6826046; doi:10.1038/s41378-019-0099-5)
Supplement: Supplementary file 3 — Supplemental Material File [file 41378_2019_99_MOESM3_ESM.docx]

# Supplementary Information

# Single-cell protein profiling in microchambers with barcoded beads

# Authors: Lucas Armbrecht^1^, Rafael S. Müller^1^, Jonas Nikoloff^1^ & Petra S. Dittrich^1^

# ^1^Department for Biosystems Science and Engineering, ETH Zurich, Mattenstrasse 26, Basel, 4058, Basel, Switzerland

**Master mould fabrication.** We fabricated the silicon master moulds for subsequent PDMS chip replication through photolithography in a standard cleanroom environment. To account for shrinkage, the structures on the two masks for the fluidic layers were enlarged by 1.6% of their desired size (all masks are appended as supplementary files). During fabrication, SU-8 3025 photoresist (Microchem, Woburn, MA, USA) was spin-coated onto two 4″ silicon wafers (SI-Mat, Kaufering, Germany) to achieve a final height of 20 μm. The resist was soft-baked and exposed to UV light (i-line, 160 mJ cm^−2^) with an MA-7 mask aligner (Süss Microtec, Garching, Germany) through the corresponding foil masks (Supplementary CAD file SF1) for the control layer and the first fluid layer (Selba S.A., Switzerland). The fluid layer master was thereafter spin-coated with a second 15 μm high SU-8 3025 layer, soft-baked and exposed through a second foil mask to pattern the magnetic capture sites. Subsequently, both master moulds underwent a post-exposure bake (5 min at 65ºC and 12 min at 95ºC), were developed for 4 min in mr-Dev 600 developer (Micro Resist Technology GmbH, Berlin, Germany), and hard-baked at 160ºC for 45 min using temperature ramps of +1.5 and -1.5ºC per min. Finally, the master moulds were silanized with *1H,1H,2H,2H*-perfluorodecyltrichlorosilane (ABCR, Karlsruhe, Germany) overnight and PTFE-coated by spin-coating 0.1% poly[4,5-difluoro-2,2-bis(trifluoromethyl)-1,3-dioxole-*co*-tetrafluoroethylene] solution in FC-40 fluorinated oil (both from Sigma-Aldrich, St. Louis, MO, USA) to simplify the release of PDMS.

**Cell culture.** Michigan cancer foundation 7 (MCF-7), SK-BR-3, and human embryonic kidney (HEK-293T) cells were grown in Dulbecco's Modified Eagle Media (DMEM, Thermo Fisher Scientific, Waltham, MA, USA) with 4.5 g L^−1^ D-glucose supplemented with 10% fetal bovine serum, 1% L-glutamine and 1% penicillin–streptomycin at 37°C in a 5% CO_2_ atmosphere (all Thermo Fisher Scientific, Waltham, MA, USA). For experiments, the cells were trypsinized (Trypsin EDTA, Thermo Fisher Scientific, Waltham, MA, USA), harvested at a concentration of ~10^6^ cells per mL in DMEM, and stained with 1 μM Calcein-AM or 1 drop of NucBlue (both Thermo Fisher Scientific, Waltham, MA, USA) for 30 min at 37°C on a MACSmix tube rotator (12 rpm, Miltenyi Biotec, Bergisch Gladbach, Germany). Afterwards, the cells were washed twice with DMEM solution and stored on ice until use for a maximum of 4 h.

For experiments with *E.coli*, LB agar and LB broth were both prepared according to the suppliers specifications. 24 h before each test, a single GFP transfected *E.coli* MG1655 bacteria colony was harvested from an agar plate that was cultured overnight and transferred to 2 mL lysogeny broth (LB) medium containing 50 ppm kanamycin sulphate (Thermo Fisher Scientific, Waltham, MA, USA). They were then incubated at 37ºC on a shaker at 200 rpm until the optical density (OD) reached a value of 1. For each test, 20 μL bacterial cell culture samples were diluted 100 times in LB medium (Sigma-Aldrich, St. Louis, MO, USA) followed by another incubation for 3 h to 4 h.

Yeast cells (*K. phaffii*) from cryo-stocks were mixed with yeast extract–peptone–dextrose (YPD) medium (Sigma-Aldrich, St. Louis, MO, USA) in a 1:20 ratio and incubated for 24 h at 30°C at 220 rpm under constant agitation. Prior to the experiments, yeast cells were diluted 1:2 in YPD medium and incubated for 30 min with 50 µM Calco Fluor White (Sigma-Aldrich, St. Louis, MO, USA) under constant rotation.  **Table S1:** RNA-expression patterns of the three investigated cell lines as listed on www.proteinatlas.org. All values are given in transcripts per million (TPM). As the RNA content between individual cells varies substantially, the number of messenger RNA (mRNA) molecules in single mammalian cell can only be approximated to on average 360,000 molecules per cell. This estimate can be used to calculate the number of mRNA molecules of the listed targets.

| Target | MCF-7 | SK-BR-3 | HEK-293T |
| --- | --- | --- | --- |
| GAPDH | 9457.8 | 7833.7 | 2834.2 |
| *Gal-3* | 118.2 | 0.5 | 2.9 |
| *Gal-3bp* | 44.9 | 1216.4 | 272.4 |


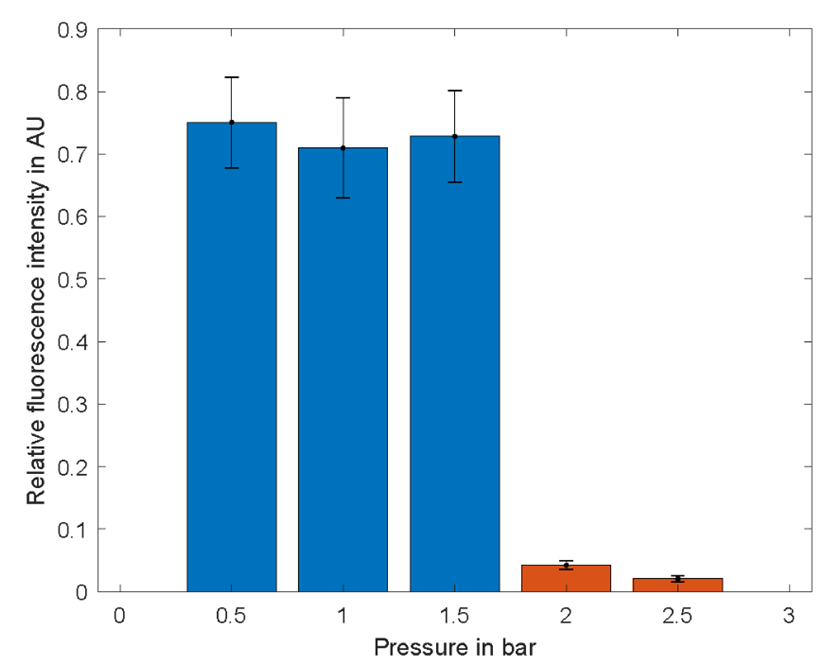


**Figure S1:** The pneumatic valves on the chip are completely closed at pressures above 2 bar, where no influx of fluorescein solution was observed during 4 hours of closure with continuous flow in the surrounding regions.


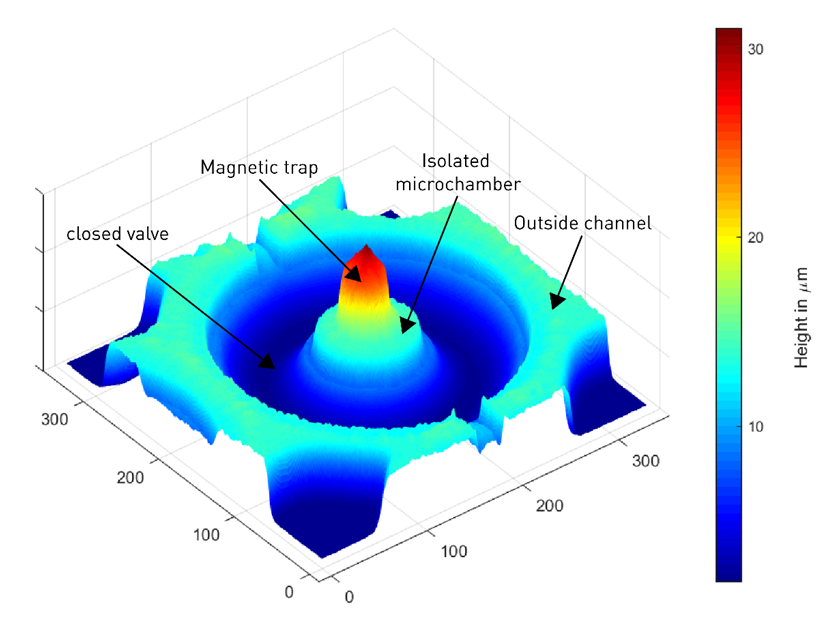


**Figure S2:** 3D plot of a microfluidic chamber created by closing the doughnut shaped pneumatic valve. This representation is generated by combining data of the channel and trap height as measured using profilometry and fluorescent images of microchambers filled with 50 µM FITC solution in DI water. First, the fluorescent signal at the locations of trap and channel were referenced to the absolute heights of the structures. Then, the volume integral of the resulting chamber profile was calculated to yield the final chamber volume of 152 pL. The chamber volume can also be approximated by two cylindrical shapes, resulting in 176 pL. difference between theoretical and actual chamber volume is resulting from PDMS deformation once pressure is applied to the valves, which decreases the actual chamber size to 86% of the original designed volume.

**
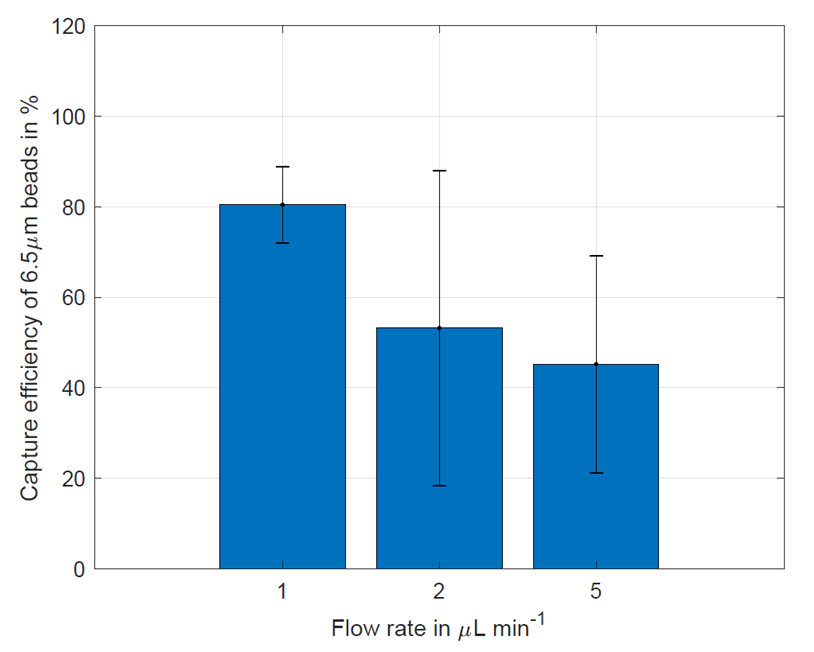
**

**Figure S3:** Flow rate dependency of the capture of 6.5 µm Luminex microparticles from 5 µL of the bead suspension, when withdrawn through the microfluidic channel at the indicated flowrates.


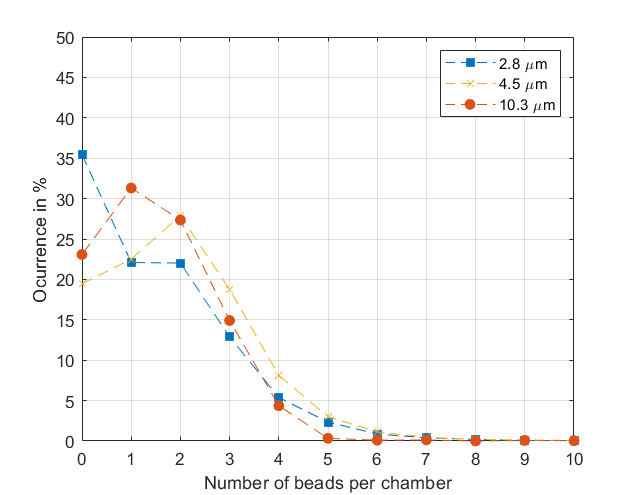


**Figure S4:** Number of beads trapped per chamber in dependence of different bead sizes. In difference to Figure 2b, all solutions were adjusted to the same particle concentration (10^7^ particle per millilitre) instead of a fixed mass concentration.


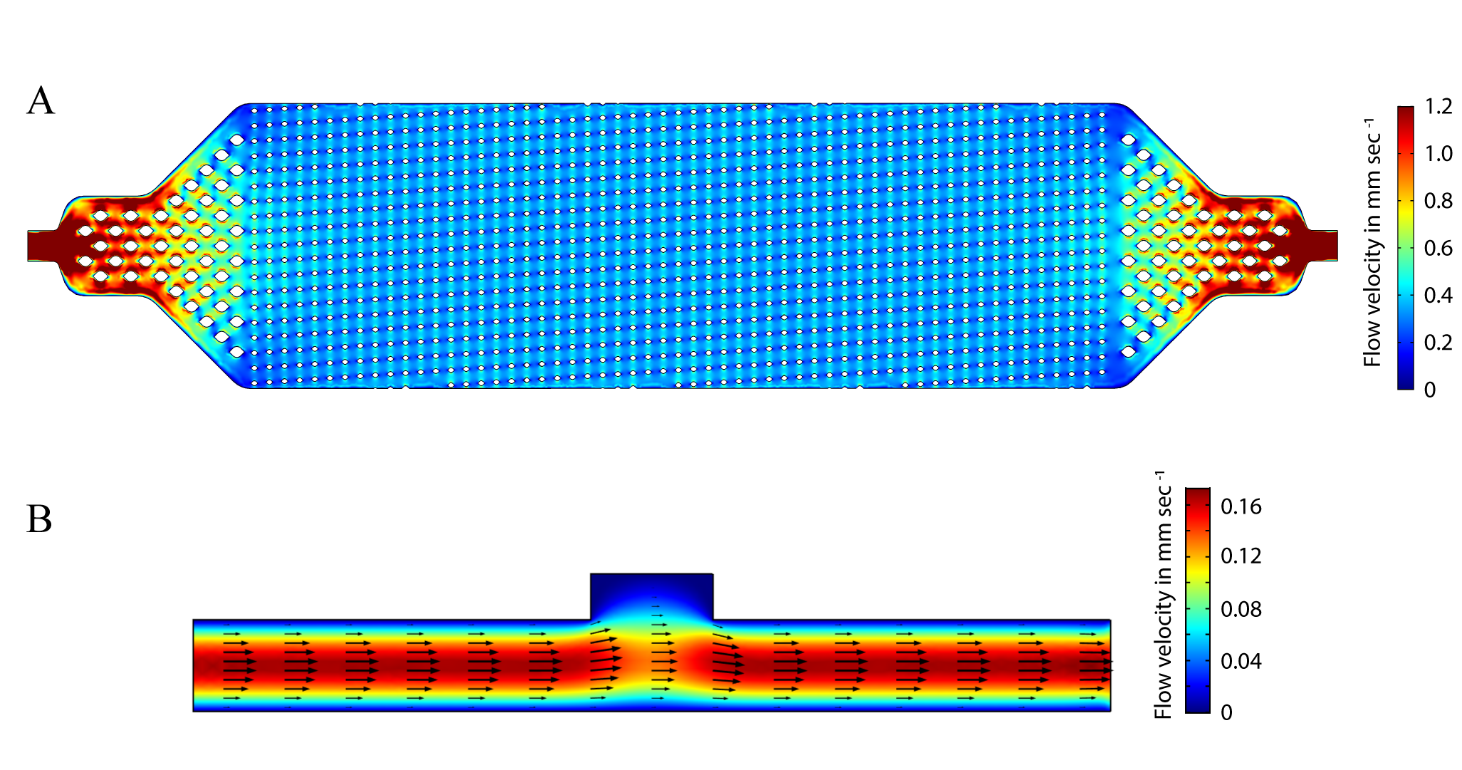


**Figure S5:** Simulation of the fluid flow in the complete microfluidic channel (the maximum width of the microfluidic channel is 6 mm. The design creates virtually homogeneous fluid flow throughout the chamber array (A). Flow in the channel cross section passing one magnetic capture site (B). The mean flow velocity into the chip was fixed to 5 µl min^-1^. The reduced flow in the top part guarantees retention of trapped particles.**
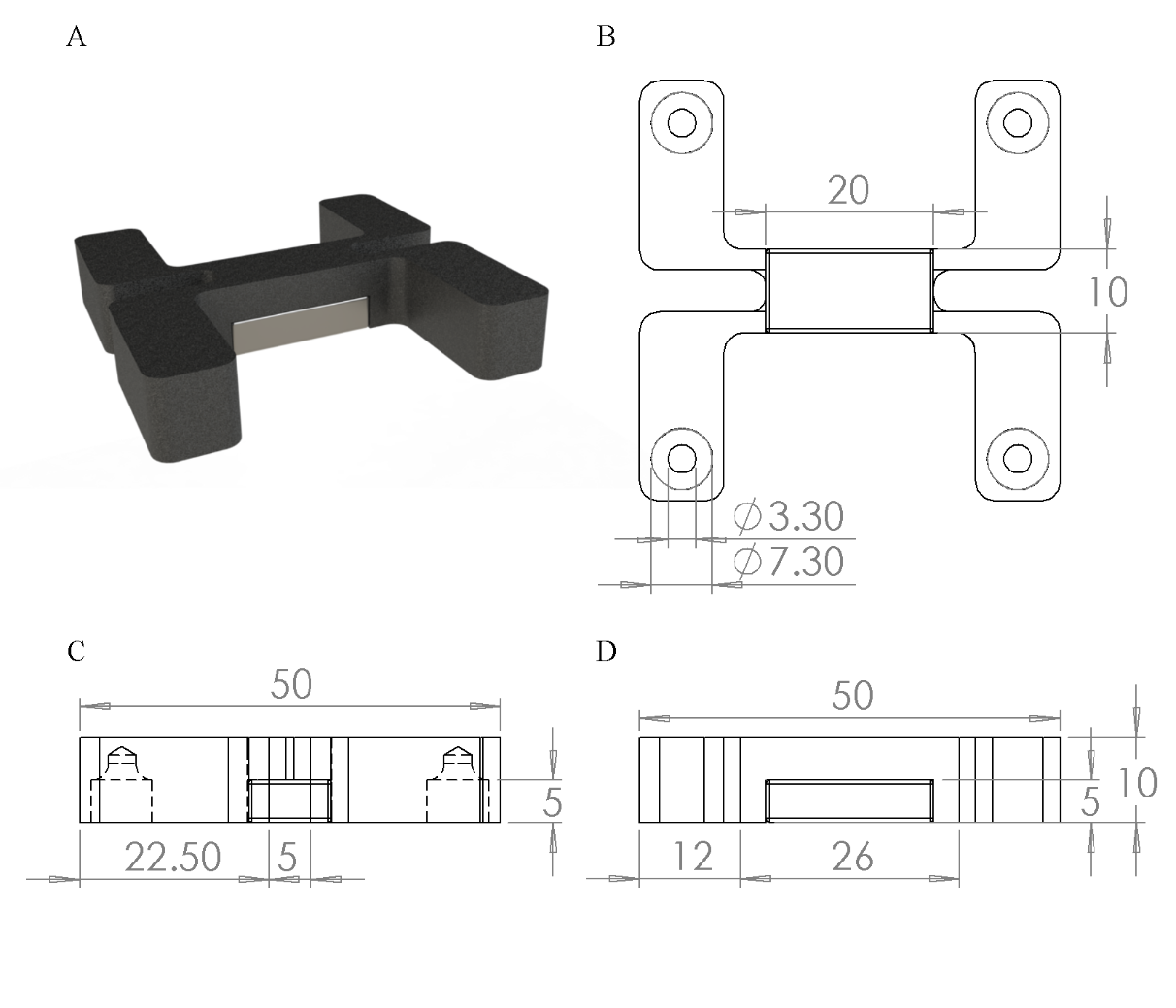
**

**Figure S6:** (A) Sketch of the device for magnetic bead actuation using an incorporated permanent magnet. (B, C, D) Technical drawing of the magnetic holder from bottom, left, and front view. All units are given in mm. For improved stability, this holder was fabricated in aluminium and anodized to yield a black surface colour. This reduces background signals when imaging with the magnetic holder mounted onto the chip.
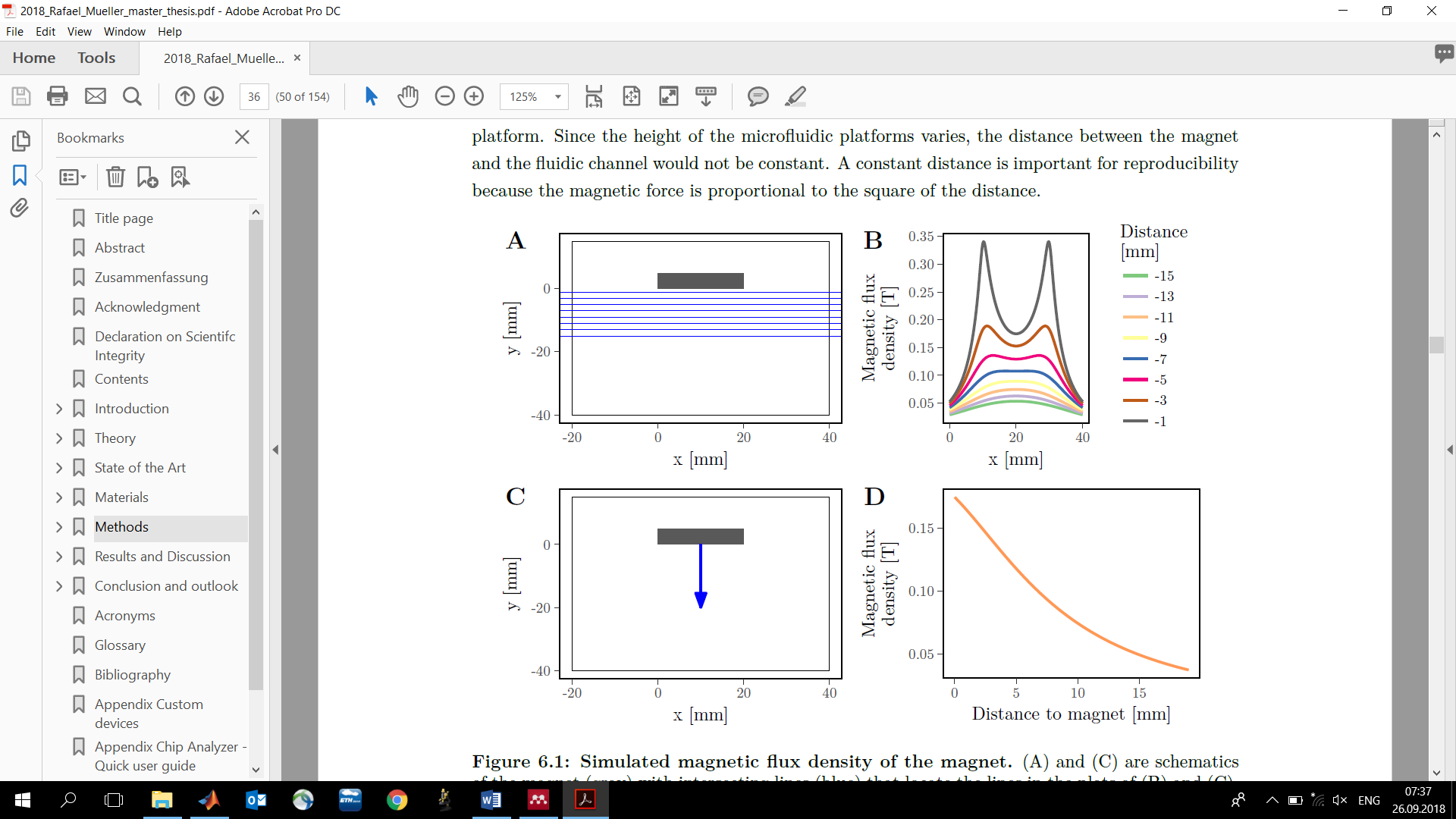


**Figure S7:** Comsol Multiphysics simulations of the magnetic field in distance of the permanent magnet. (A) and (C) are schematics of the magnet (gray) with intersecting lines (blue) that indicate directions of the magnetic flux simulations in (B) and (C), respectively. (B) The magnetic flux parallel to the magnet in distances from the magnet ranging from 1 mm to 15 mm. We found that a distance larger than 7 mm generates a homogeneous field strength along the channel while smaller distances generate maxima close to the inlet and outlet of the chip. As the absolute magnetic field strength decreases with the distance (D), the optimal distance between channel and magnet was found to be between 7 and 9 mm.


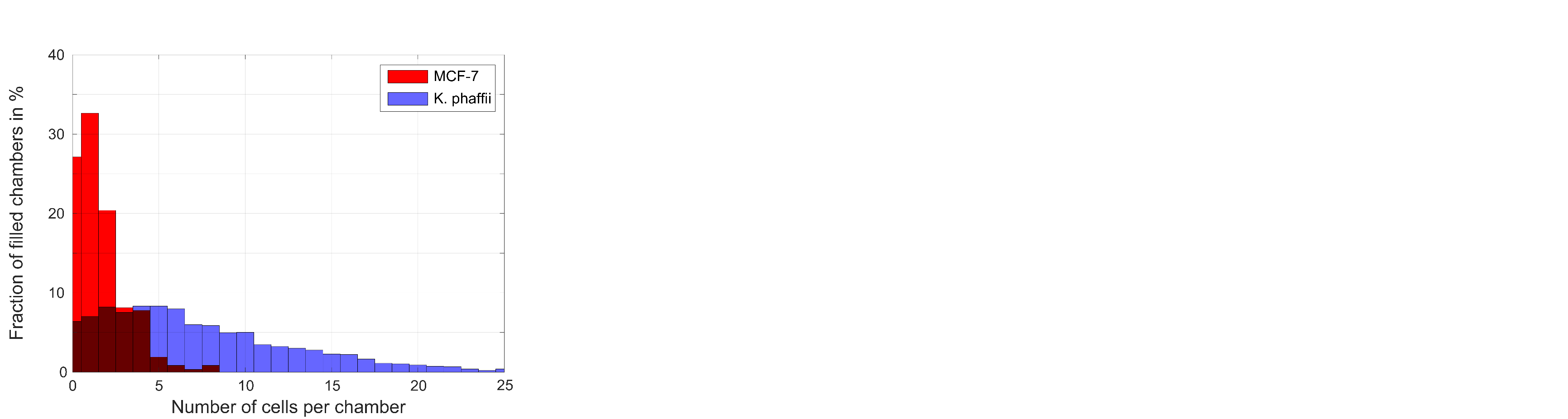


**Figure S8:** As *K. phaffii* yeast cells grow in agglomerates of several cells, the capture of these cells yields a broad distribution of cell numbers per chamber. Mammalian breast cancer MCF-7 cells have an epithelial origin and can form clusters as well. The clusters however consist of fewer cells such that the capture distribution is narrower and 35% of the chambers are occupied by a single cell.


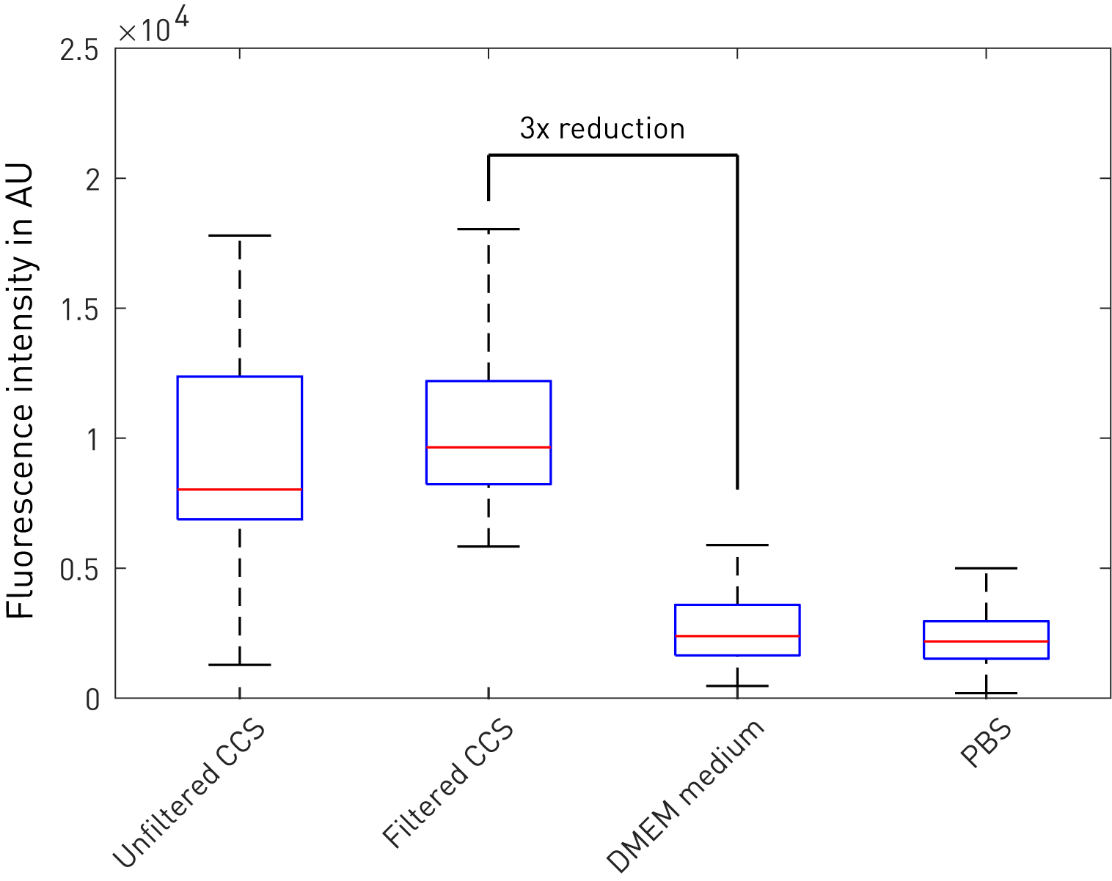


**Figure S9:** Background signals of different media. When beads get in contact with the sample (cell solution or cell supernatant), target molecules from the sample solution can bind to the primary antibodies on the bead surface. This leads to an increased background signal that interferes with the signal from the tested single cell. In this graph, the resulting background signals for the Gal-3bp assay using SK-BR-3 cell culture supernatant (CCS) and different media as samples are shown. Introducing beads after cell capture and washing prevents contact between beads and CCS and hence, reduces the background signal by a factor of three.


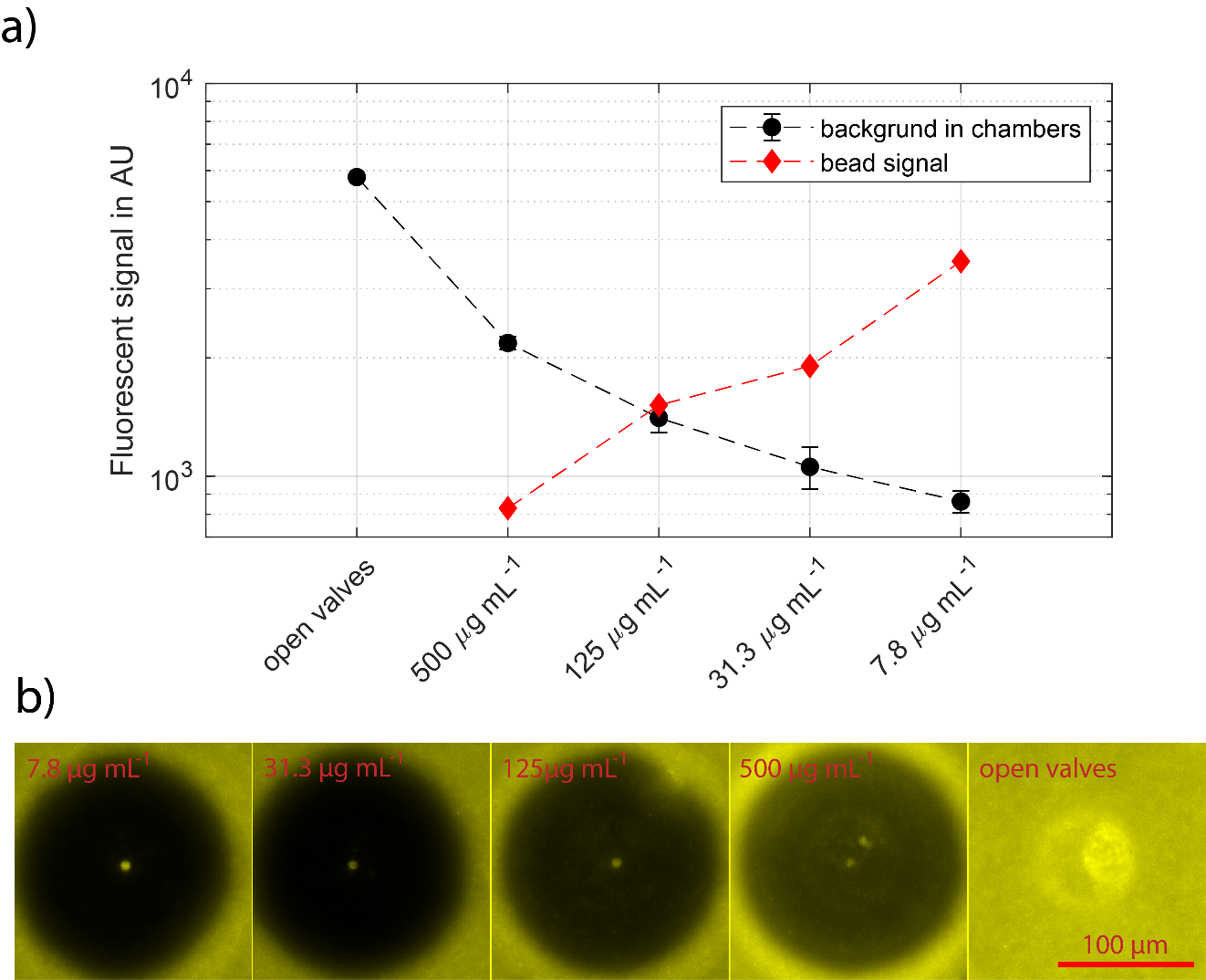


**Figure S10:** The GAPDH assay shows lower signals for high target concentrations similar to the high-dose hook effect. Here, we used extremely high concentration of GAPDH to understand this finding. In a) we observe that the signal on the bead decreases while the background signal rises for GAPDH concentrations in the high microgram per milliliter range. B) The increase in the background fluorescence with increasing GAPDH concentration is clearly visible as well as the decrease in the signal at the bead is also clearly visible in the fluorescence images.


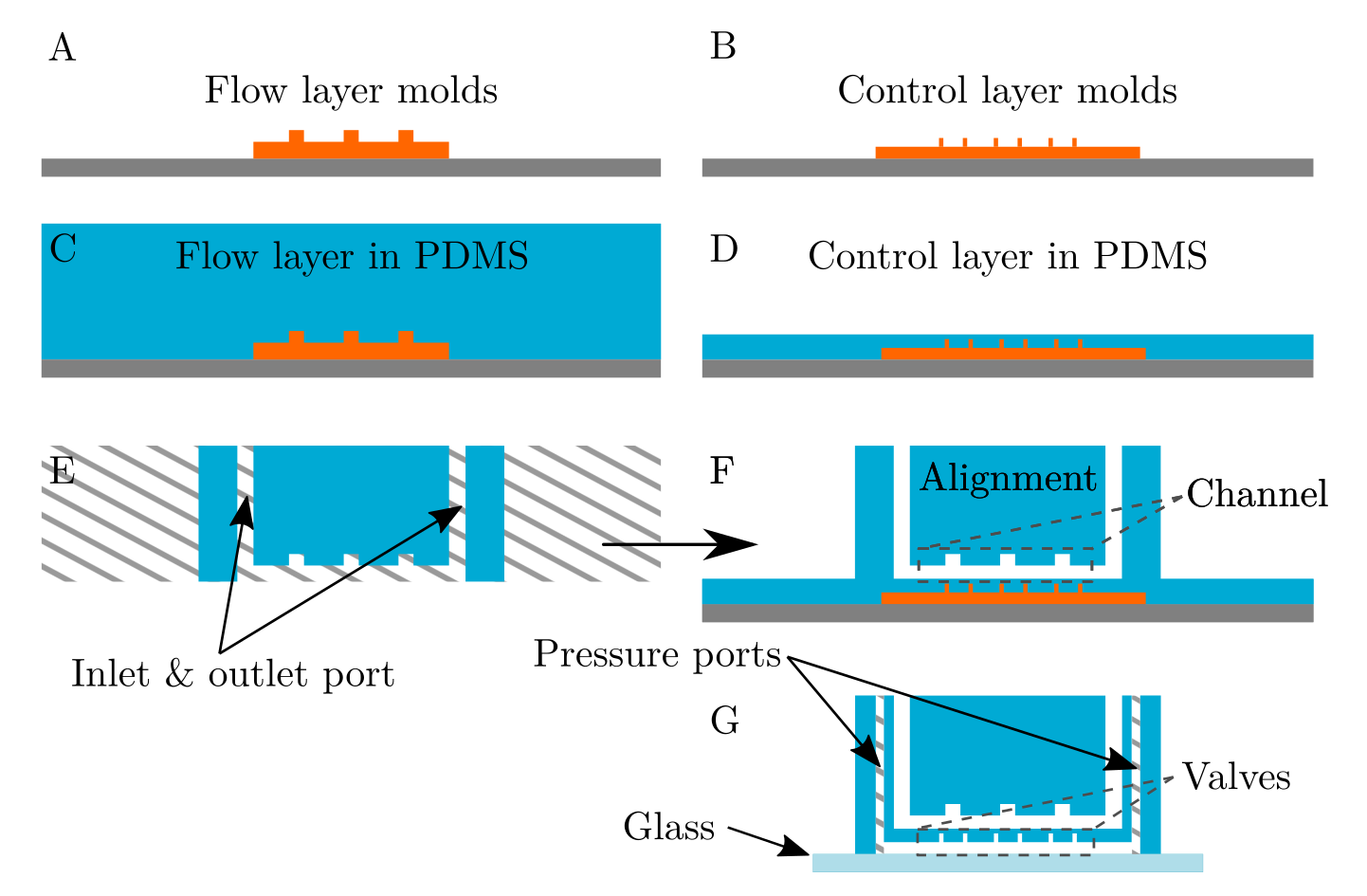


**Figure S11:** Schematic of microchip fabrication (not to scale). (A, B) Two silicon master moulds (gray: silicon, orange: photoresist) for the flow layer and the control layer, respectively, are fabricated in a cleanroom facility. (C, D) PDMS monomer and curing agent are mixed in a 10:1 ratio and deposited as a ~5 mm thick layer on the flow layer structures (C). Similarly, PDMS is spin-coated onto the control layer to form an approximately 30 µm thin layer (D). (E) After curing the parts and removing the PDMS from the mould, the flow layer is cut to size and fluidic inlet and outlet holes are punched. (F) Next, the control layer is covered by dipping into a thin film of curing agent (not shown) and both parts are aligned and bonded together. (G) After bonding, the two-layer structure are peeled off the control layer master mould and all control ports are punched. Finally, the chip is bonded to a glass cover slip.


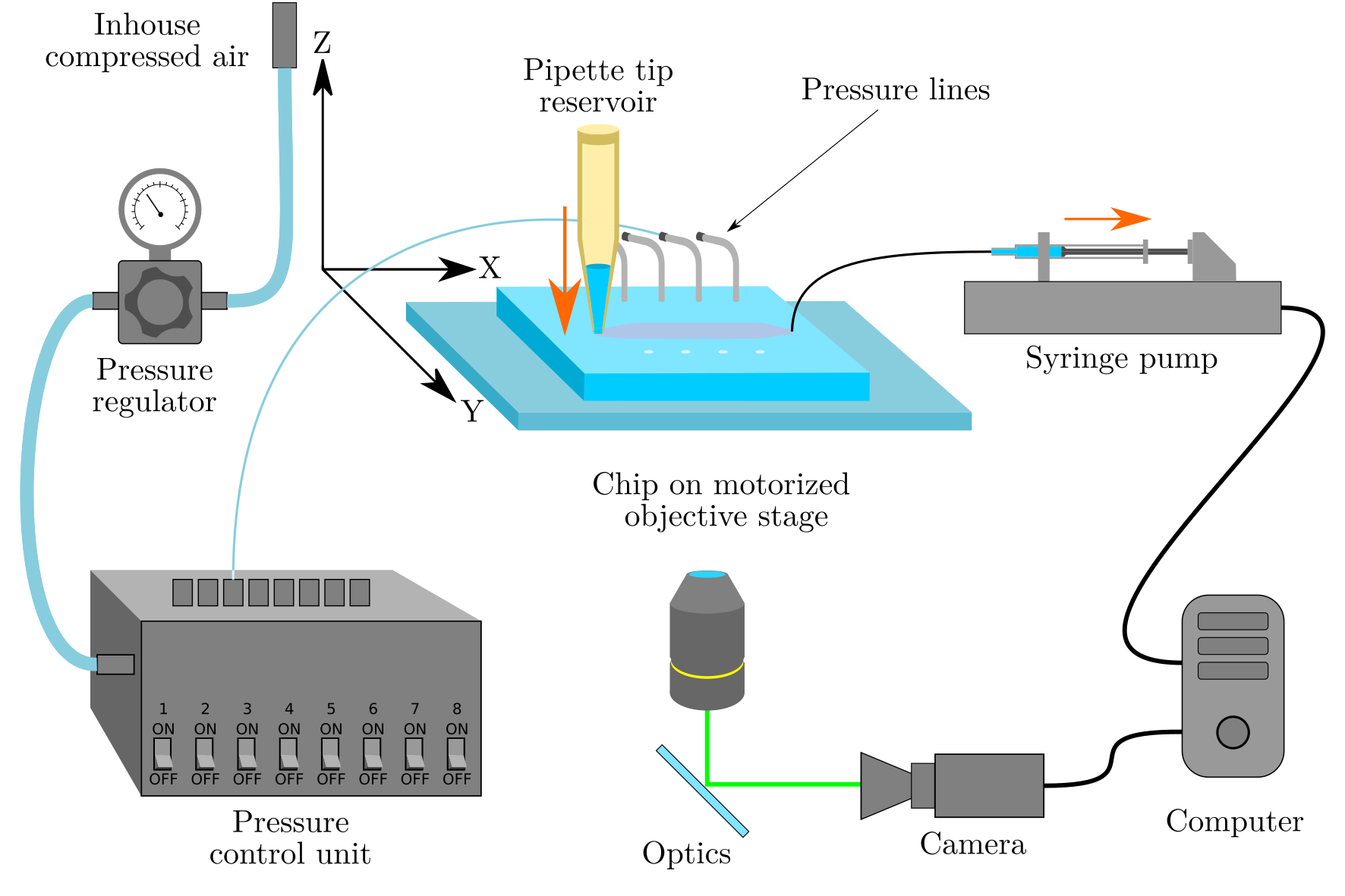


**Figure S12**: Schematic representation of the experimental setup. The microfluidic chip is fixed on the automated stage of the fluorescence microscope. All eight pressure lines are connected to one pressure control unit that opens and closes all pneumatic valves. The absolute pressure for all valves is set with an additional pressure regulator. Liquids from the pipette tip are aspirated by a syringe pump. The stage, the light sources and the camera are controlled with the Nikon NIS Elements V 5.02 imaging software (Nikon, Tokyo, Japan) while the syringe pump is controlled with the neMESYS (Cetoni GmbH, Korbußen, Germany) user interface.


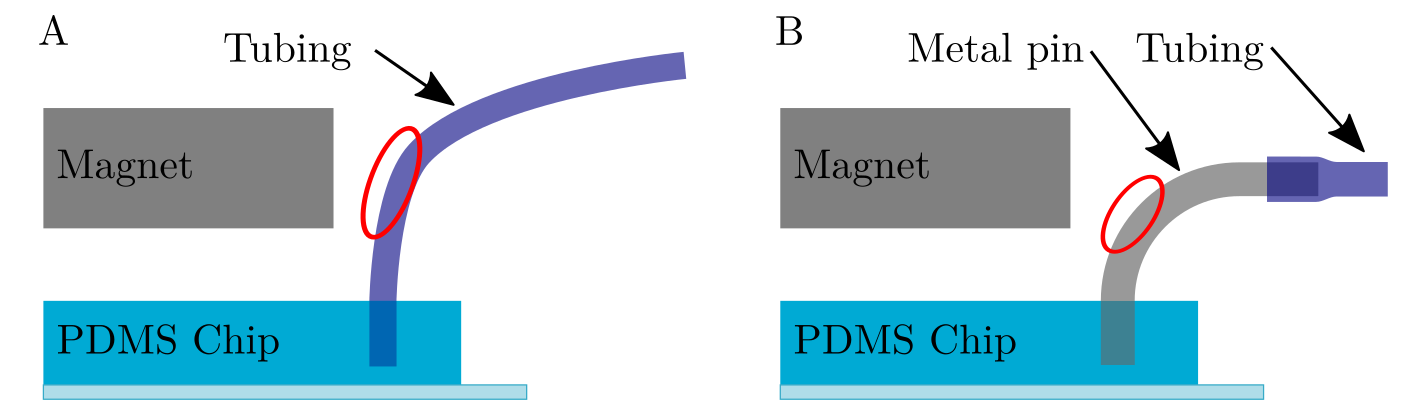
**Figure S13**: A stainless steel pin facilitates the connection from the syringe pump to the chip and simultaneously acts as a Faraday cage that shields the flow to and from the chip from external magnetic fields. This reduces undesired bead accumulation in the location of closest proximity between beads and magnets which is indicated in red.

**Supplementary files**

**Supplementary Video SV1:** Washing of microchambers observed at 1 µL min^-1^. The time is referenced to the moment, the valve was de-pressurized. Images were acquired with a Hamamatsu Orca Flash camera on an automated Nikon Ti2 microscope (40X objective, NA=0.45).

**Supplementary CAD File:** The .dwg file incorporates the master structures for the photolithographic fabrication of the silicon master moulds. The structures of the top fluid layer are enlarged by 1.6% to compensate for shrinkage during the chip fabrication procedure.

**Software for image analysis**

The Matlab code for the data reduction GUI, explained in more detail below, is available upon request from the authors.

**General principle.** To reduce the image file size and simplify data analysis, all microchambers were extracted from the initial multidimensional *.nd2 image file with a custom software tool. First, the user selects the chip design (array of microchamber positions) through the graphical user interface (GUI). As the theoretical locations of these regions do not match the real positions in the image file, the user is asked to manually select the coordinates of two known positions. The software then transforms the given coordinate-list through scaling and rotation and crops and extracts images of all chambers from the original file.

Our cropping routine supports multiple positions, channels, z-planes, and time points, is able to perform shading correction during processing, and saves all data and the associated metadata in a *.mat file. Subsequently, data analysis is carried out with secondary Matlab scripts that sequentially detect cells and fluorescent particles in each chamber. Once detected, the number of cells per chamber as well as the eccentricity, image position, and the mean intensities of barcoded beads in all fluorescent channels are extracted. The fluorescent intensities in the two red channels (658 nm and 712 nm) reveal the barcode of the bead while the immunoassay signal is read at 560 nm. The first part used to extract all microchamber data from large multidimensional image stacks acquired with a Nikon Ti2 microscope will now be described in more detail.


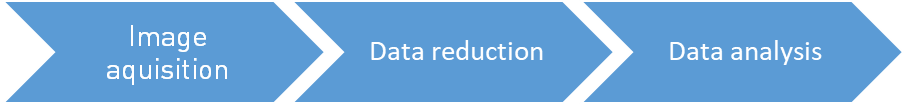


**Fundamentals.** The algorithm takes advantage of an adapted version of the “Java Bio-Formats” class to read the files into the system, the software is able to process not only “*.nd2” files, but was also tested with “*.nd” files (Linkert, M. *et al.* Metadata matters: Access to image data in the real world. *J. Cell Biol.* **189,** 777–782, 2010). Hence, it is likely that other file formats can be analysed without adapting the software as long as they are supported by the “Bio-Formats” package.


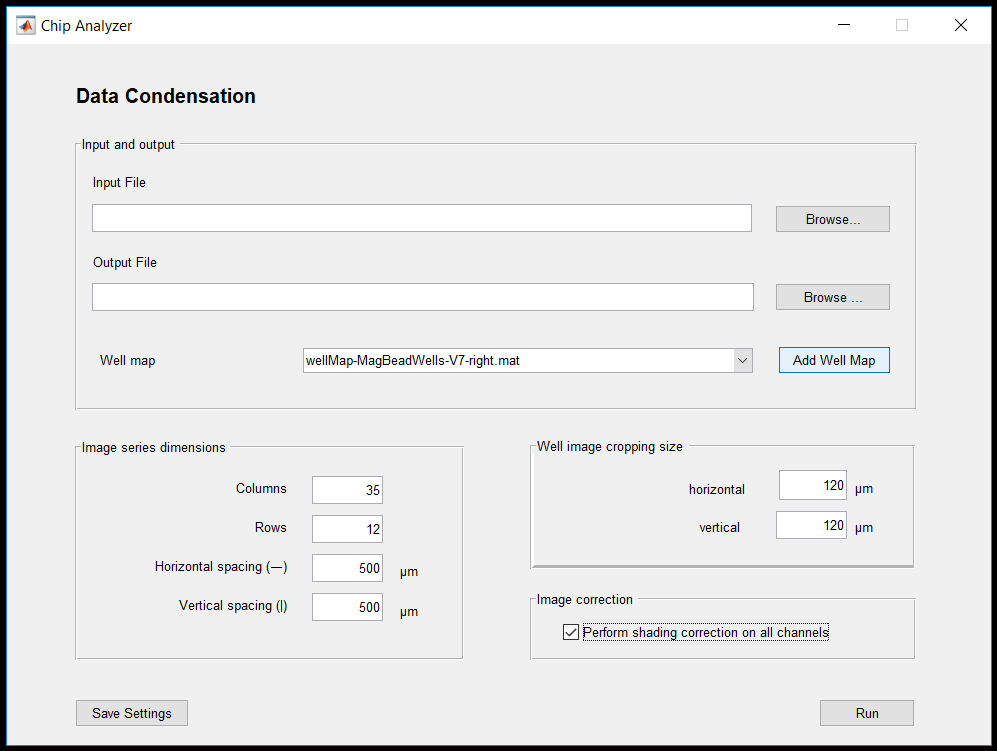
**Installation and start-up**. First, the user needs to download the entire program (attached .zip file), extract the internal folder structure, and save it in their preferred Matlab directory. Once this is done, the corresponding directory including all subfolders has to be added to the Matlab path. At this point the user is able to start the “*/src/Main.m” file (please note that the current path has to be set as the “*/src” folder to successfully run the routine and that a valid Matlab license - at least version 2017b - is required). In case of problems, the user can try running the “MainTest.m” script to evaluate the source of the error. When used in Mac OS, the font sizes of all GUIs are enlarged to compensate for differences between operating systems.

**Figure S14**: Main graphical user interface for data reduction and extraction of multiple positions from a multidimensional image file.

**Usage of the GUI.** Once the program is started, a graphical user interface should appear (see Figure S14). Here, the user can select the image file for analysis as well as the output directory to store the processed and reduced data. Additionally, there is a pop-up window to select the well-map (look-up table of “to-be-cropped” positions referenced to one another – all dimensions in this table are to be given in micrometres). In case the chip design is not listed the user can click a button to add a new well map. Additional well maps need to be provided as an Excel file with two columns holding the x and y positions of all positions of interest in micrometres. An example with three positions would be:

| **Excel line** | **Column A (X-position)** | **Column B (Y-position)** |
| --- | --- | --- |
| **1** | 0 | 0 |
| **2** | 0 | 500 |
| **3** | 0 | 1000 |
| **4** | 500 | 1000 |
| **5** | 500 | 500 |
| **6** | 500 | 0 |

In the above example, three positions would be extracted from the image series. When the appropriate well map has been defined and selected, some general image information is required. This includes the number of rows and columns of the image series, the distance between individual rows and columns, and the desired size of the final cropped images. When this is done, the user can run the script.

If the metadata and the inserted image parameters fit, the user is led to define the position of the first chamber in the well map by opening a user interface for this purpose. Here, the user can switch between channels, time points, positions, or *z*-planes if necessary and finally selects the first position. Once selected, the user is guided to the selection of one more position. This is the position of the exact chamber that is furthest from the first chamber. Again, the user can then move through channels, time point, *z*-stacks and positions if necessary to select the second position. As default, the software assumes image acquisition serpentine loops. Hence, it opens the top left image for selection of the first chamber and thereafter the bottom right image for selection of the last chamber of interest. When both positions are selected, the software starts its internal routine to extract all positions from the image file using a projection of the transformed original well map (scaling and rotation). In comparison to feature recognition, this workflow turned out to be more robust and works independently of the chip design (see ).

**Output file.** The output file is a Matlab “*.mat” file that golds two variables named “glbMeta”, and “well”. The first is a cell array that provides all metadata such as pixel sizes, locations and timestamps as well as process parameters that indicate if shading correction has been performed. Besides, the second cell array “well” includes all images of cropped positions in a well-defined and easily accessible form. Images can be retrieved with *well{1, position}{time point, channel}*. The returned image is 2D in cases where a single *z*-plane was imaged, or 3D matrix when *z*-stacks were acquired. This notation allows the use of simple while-loops to run complex analysis routines on all images from one experiment.
